# Systematic identifiability testing for unambiguous mechanistic modeling – application to JAK-STAT, MAP kinase, and NF-*κ*B signaling pathway models

**DOI:** 10.1186/1752-0509-3-50

**Published:** 2009-05-09

**Authors:** Tom Quaiser, Martin Mönnigmann

**Affiliations:** 1 Institute for Heat and Fuel Technology, Technical University Braunschweig, D-38106 Braunschweig, Germany; 2 AVT – Process Systems Engineering, RWTH Aachen University, D-52064 Aachen, Germany

## Abstract

**Background:**

When creating mechanistic mathematical models for biological signaling processes it is tempting to include as many known biochemical interactions into one large model as possible. For the JAK-STAT, MAP kinase, and NF-*κ*B pathways a lot of biological insight is available, and as a consequence, large mathematical models have emerged. For large models the question arises whether unknown model parameters can uniquely be determined by parameter estimation from measured data. Systematic approaches to answering this question are indispensable since the uniqueness of model parameter values is essential for predictive mechanistic modeling.

**Results:**

We propose an eigenvalue based method for efficiently testing identifiability of large ordinary differential models and compare this approach to three existing ones. The methods are benchmarked by applying them to models of the signaling pathways mentioned above. In all cases the eigenvalue method proposed here and the orthogonal method find the largest set of identifiable parameters, thus clearly outperforming the other approaches. The identifiability analysis shows that the pathway models are not identifiable, even under the strong assumption that all system state variables are measurable. We demonstrate how the results of the identifiability analysis can be used for model simplification.

**Conclusion:**

While it has undoubtedly contributed to recent advances in systems biology, mechanistic modeling by itself does not guarantee unambiguous descriptions of biological processes. We show that some recent signal transduction pathway models have reached a level of detail that is not warranted. Rigorous identifiability tests reveal that even if highly idealized experiments could be carried out to measure all state variables of these signaling pathways, some unknown parameters could still not be estimated. The identifiability tests therefore show that the level of detail of the investigated models is too high *in principle*, not just because too little experimental information is available. We demonstrate how the proposed method can be combined with biological insight, however, to simplify these models.

## Background

Several large and detailed mathematical models for signal transduction pathways exist in the literature. Lipniacki et al. [1] model the NF-*κ*B pathway using 15 state variables and 29 parameters. Yamada et al. [2] introduce a system of ordinary differential equations that describe the JAK-STAT pathway with 31 state variables and 52 parameters, and Schoeberl et al. [3] describe the EGF pathway with a model that comprises 103 variables and 98 parameters. Models of this kind can provide a concise and unambiguous representation even of very complex signaling pathways. However, since usually some of their parameters are not known, these models pose demanding parameter estimation problems. Two fundamental problems must be considered in this context: 1) the larger the number of unknown parameters in a model, the larger the amount of quantitative data necessary to determine meaningful values for these parameters. 2) Even if appropriate experimental data are available, model parameters may not be uniquely identifiable [4]. Ultimately, reliable predictive mathematical models can only be created after addressing these two problems.

Parameter identifiability has been paid little attention to in the recent systems biology literature. Exceptions exist, however. Swameye et al. [5] carefully check the identifiability of their JAK-STAT model. The authors propose a small model (four state variables and six parameters) and demonstrate that the model provides quantitatively reliable predictions despite its small size. In the field of HIV/Aids modeling differential algebraic techniques have been used to prove identifiability [6-8] for models of similar size. Other examples can be found in [9,10].

The problem of testing identifiability amounts to answering the following question: given a mathematical model of a system together with system input and output data, are the model parameters uniquely determined? The identifiability tests investigated here can be carried out before experimental data are available. To this end a model is used first to generate simulated data. Subsequently, it can be checked whether the model parameters are uniquely defined by the simulated data. Only if identifiability can be assured for the model and the simulated data it is reasonable to continue with lab experiments, identifiability tests with experimental data, and, eventually, parameter estimation. In the present paper we exclusively use simulated data.

Several notions of identifiability exist. We attempt a brief and informal summary of the essential ideas here. Stricter terminology is introduced in subsequent sections. Essentially, a model is called globally identifiable if a unique value can be found for each model parameter such that the model reproduces the measured or simulated output data. If, in contrast, a finite number of points in the model parameter space can be found, for which the model reproduces the output data the model is called locally identifiable. Finally, if an infinite number of parameter values exists that reproduce the model input-output behavior, the model is considered to be unidentifiable. Independently of these three notions we need to distinguish between at-a-point identifiability on the one hand and structural identifiability on the other hand [11,12]. These two concepts distinguish two classes of methods for identifiability testing from one another. Methods for at-a-point identifiability testing can only be applied if candidate values for the model parameters are known a priori. This situation arises, for example, when parameter values for a model have already been published in the literature, as is the case for the signaling pathway models investigated here. In contrast, all mathematically and biologically possible parameter values must be considered as candidate values, if no information on the model parameter values is known a priori. In this case we speak of structural identifiability testing.

Several techniques exist for analyzing structural identifiability. These methods are based on power-series expansion [13], transfer function analysis [14], differential algebra [6,12,15], interval arithmetics [16], state isomorphisms [17-19] or semi-infinite programming [20,21]. These methods are, however, either restricted to linear models or to models with less than 10 states and parameters in the nonlinear case [22].

For large nonlinear models only methods for testing local at-a-point identifiability are feasible. A number of methods to test local at-a-point identifiability have been proposed in the literature. Some of these aim at determining the largest subset of identifiable parameters [23,24], other methods are tailored to finding the unidentifiable parameters [25,26], or to finding parameters that do not affect the input-output behavior of the model [27,28]. All these methods are based on the sensitivity matrix of the model responses (for details see section Methods for local at-a-point identifiability testing). In contrast to the approaches mentioned so far the method introduced in [29] does not depend on sensitivity information. This method repeatedly estimates parameters with randomly chosen start values and extracts dependencies between parameters with a statistical method. The data used for the parameter estimation steps is created by simulating the model at a nominal parameter point. The approach does not belong to the class of methods for at-a-point identifiability testing, since it is delocalized by using the above mentioned random multistart approach for parameter estimation. Consequently, the method is more rigorous than the discussed at-a-point identifiability tests, but not as rigorous as the structural methods.

The examples treated here turn out not to be locally at-a-point identifiable, therefore it is not reasonable to consider stricter concepts of identifiability. In the sequel the term identifiability will refer to local at-a-point identifiability if not noted otherwise.

We compare the methods for identifiability testing introduced by Yao et al. [24], Jacquez and Greif [25], and Degenring et al. [27], which for ease of reference we refer to as the orthogonal method, the correlation method, and the principle component analysis (PCA) based method, respectively. Specifically, we would like to establish which of these approaches is the method of choice for identifiability testing of large signal transduction pathway models. We omit the method published by Brun et al. [23] due to its combinatorial complexity. Similarly, the method proposed by Hengl et al. [29] is omitted, because it proved to be too computationally demanding for the examples treated here. The method was tested with default settings. When applied to the smallest of the models treated here, the wall clock computation time was about 15 hours in contrast to wall clock computation times of less than a minute for the methods compared here. When applied to the JAK-STAT model, the method proposed by Hengl et al. [29] did not finish within a week. It was therefore not included in the comparison. Finally, we note an interesting approach has only very recently been proposed by Chu and Hahn [30]. Since this approach solves a problem that is similar to, but after all different from, the identifiability tests addressed here, it is not included in the comparison. All results are compared to results found with our own method [31], which we refer to as the eigenvalue method for short. The eigenvalue method is an extension of an approach published by Vajda et al. [26]. We note the eigenvalue method has independently appeared in a recent paper by Schittkowski [32].

This paper has three contributions: 1) we reveal that three well established models of signal transduction are not identifiable, and demonstrate how results from identifiability studies can be used to simplify these models. 2) We suggest an efficient numerical method for identifiability testing of large nonlinear systems of ordinary differential equations in general, and 3) we compare our method to three previously published methods.

This paper is structured as follows. We start by reviewing the theory of identifiability and introduce the four methods for local at-a-point identifiability testing compared here. Subsequently, the three pathway models used in the case studies are introduced and each of the models is analyzed with each of the methods. Insight from this analysis is used to simplify the models.

## Methods

In this section we will first introduce the mathematical system class and give a more concise definition of identifiability. We focus on local at-a-point identifiability and describe the four methods for testing local at-a-point identifiability compared here. Finally, we summarize the three signaling pathways and the corresponding models.

### System Class

The concept of identifiability applies to a large system class. Here we treat models of the form

(1)

since many biological signaling pathways can be represented by systems of this form. In equation (1) , ,  and  denote the state variables, the parameters, the inputs, and the outputs of the dynamical system, respectively. A biological parameter, for example a kinetic constant, corresponds to a component *p*_*k *_of the parameter vector. The functions *f *and *h *map from an open subset  onto  and , respectively, and are assumed to be smooth. Note both a lab experiment and a simulation are uniquely defined by the initial conditions *x*(0) = *x*_0 _and the values of the inputs *u*(*t*) from *t *= 0 to the final time *t *= *t*_*f*_. Both an experiment and a simulation result in values of the outputs *y*(*t*) at successive points  in time

(2)

where *n*_*t *_denotes the number of measurements or stored simulation results, respectively. In the present paper, output data of the form (2) is obtained from simulations with models that have been adjusted to experimental data by other authors before [1-3].

The solution of equation (1) that results for a particular choice of initial conditions *x*_0_, parameters *p*, and inputs *u*(*t*) is denoted by

(3)

The solution of equation (1) and its derivatives with respect to the parameters are computed with the integrator DDASPK [33]. The output behavior of the model is given by the response function

(4)

### Concept of identifiability

Here we summarize the notions of identifiability as necessary for the paper (see [4] for a review). Assuming that the inputs *u*(*t*), the initial conditions *x*_0_, and the measurement times *t*_*i *_are given, the different notions of identifiability can be defined as follows.

*Definition 1: *The parameter *p*_*k *_of the model (1) is called globally structurally identifiable if, for all admissible values  and all ,

(5)

implies

(6)

*Definition 2: *The parameter *p*_*k *_of the model (1) is called locally structurally identifiable if, for all , there exists a neighborhood  such that for all , equations (5) imply equation (6).

The parameter vector *p *is called globally structurally or locally structurally identifiable if all its components *p*_*k *_are globally structurally or locally structurally identifiable, respectively. Global and local at-a-point identifiability are defined as follows.

*Definition 3: *Let  be a point in the parameter space of the model (1). The parameter *p*_*k *_of the model (1) is called globally at-a-point identifiable at the point p* if, for all , equations (5) imply equation (6).

*Definition 4: *Let  be a point in the parameter space of the model (1). The parameter *p*_*k *_of the model (1) is called locally at-a-point identifiable at the point p* if there exists a neighborhood  such that for all , equations (5) imply equation (6).

The following subsection will introduce the four methods for at-a-point identifiability testing compared in this work.

### Methods for local at-a-point identifiability testing

The compared numerical methods for local at-a-point identifiability testing are based on the sensitivity of the model outputs at discrete time points *t*_*k*_, *k *∈ {1,..., *n*_*t*_}, with respect to the parameters. The sensitivity information is stored in the *n*_*y *_*n*_*t *_× *n*_*p *_dimensional sensitivity matrix *S*. *S *is a block matrix that consists of time dependent blocks *s*(*t*_*i*_) of size *n*_*y *_× *n*_*p *_[34]:

(7)

The entries of *s*(*t*_*i*_) are called sensitivity coefficients. For a nominal parameter vector p*, given *x*_0_, and fixed time *t*_*k *_with *k *∈ {1,..., *n*_*t*_}, they are defined as

(8)

Essentially, the sensitivity coefficients describe how sensitive the system output is to changes in a single parameter. If the model output is highly sensitive to a perturbation in one parameter we can consider this parameter to be important for the system behavior. In contrast, a parameter that has no influence on the outputs is a candidate for an unidentifiable parameter. Linearly dependent columns of the sensitivity matrix imply that a change in the system outputs due to a change in one parameter, say *p*_*j*_, can be compensated by changing some or all of the dependent parameters *p*_*k*_, with *k *≠ *j*. If dependencies exist, parameter estimation will fail or result in non-unique parameter values [26]. The correlation and orthogonal method are based on this idea.

For ease of presentation, we first give a detailed introduction to the eigenvalue method proposed here. The remaining methods are summarized briefly only, and the reader is referred to the appendix for more details. Since all methods analyze at-a-point identifiability, nominal values for the parameters are required. For the test cases treated here parameter values are available from the literature [1-3] and denoted by *p*^lit^.

#### Eigenvalue method

The eigenvalue method is similar to the method published by Vajda et al. [26]. The eigenvalue method proposed here differs from the approach proposed by Vajda and coworkers in that a manual inspection of eigenvalues and eigenvectors is not necessary here. As a result, an automatic analysis of large models becomes feasible. We first describe the eigenvalue method and subsequently describe differences to the method published by Vajda et al. [26] in more detail.

We consider the least squares parameter estimation problem that amounts to minimizing cost functions of the form

(9)

with respect to *p*, where the *y*_*i *_(*t*_*j*_) are the measured or simulated data introduced in equation (2). In Gaussian approximation, the Hessian matrix *H *of equation (9) has the entries

(10)

for *k*, *l *∈ {1,..., *n*_*p*_}. As indicated in equation (10), *H *can be expressed in terms of the sensitivity matrix *S *introduced in equation (7). *H *is a symmetric matrix and therefore its eigenvalues are real. Furthermore, *H *is positive semi-definite.

In order to study the identifiability of the system in equation (1) for given *x*_0_, *u*(*t*) and for nominal parameters  we first solve equation (1) by numerical integration and subsequently calculate *H *as given by equation (10). We set p* to parameter values taken from the literature *p*^lit ^in all calculations. All identifiability tests carried out in the present paper therefore amount to asking whether the investigated signaling models are identifiable at the literature parameter values *p*^lit^. As pointed out already in the Background section, identifiability tests of this type clarify whether a given model is reasonably detailed even before experiments are carried out. In this sense, the identifiability of a model at nominal or literature values for its parameters is considered to be a necessary condition for its practical identifiability with measured data.

Let *λ*^*j *^and *u*^*j *^denote the *j*th eigenvalue and the corresponding eigenvector of *H*, respectively. Assume the eigenvalues to be ordered such that , assume the eigenvectors *u*^*j *^to be normalized such that *u*^*jT *^*u*^*j *^= 1, and assume p* to minimize equation (9). Consider the change of *ϕ *when moving from p* in a direction *αu*^*j *^for some real *α*. Gaussian approximation yields

(11)

where the linear order is zero, since p* is is a minimum by assumption. Since *Hu*^*j *^= *λ*^*j *^*u*^*j *^and *u*^*jT *^*u*^*j *^= 1, the last equality in equation (11) holds. Equation (11) implies that *ϕ *does not change when moving from p* to p* + Δ*p *if Δ*p *= *αu*^*j *^for any eigenvector *u*^*j *^with *λ*^*j *^= 0 and any real *α*. In words, the directions *u*^*j *^corresponding to *λ*^*j *^= 0 are those directions in the parameter space along which the least squares cost function is constant. We call these directions degenerate for short. In the particular case of

(12)

where the entry 1 is in position *k*, the model is not identifiable with respect to the *k*th component . The approach proposed here will therefore remove this parameter from consecutive calculations by fixing  to . In general however, *u*^*j *^will not be of the special form (12), but *u*^*j *^will be a vector with more than one non-zero entry, therefore the choice of *k *is not obvious. In this case, we select a *k *such that , and remove the *k*th component of p* from the parameter estimation problem by fixing it to its literature value. In the examples treated below this choice of *k *turns out to be appropriate in all cases. In general, this might not be the case, however. There might be entries of , *l *≠ *k*, with an absolute value  close or equal to the maximal value . We call such entries  and the corresponding parameters co-dominant, since together with  they dominate the degenerate direction. A simple example for a system with co-dominant parameters is given in Appendix A. We will discuss the issue of co-dominant parameters for the examples treated here in the Results section.

In practical applications the smallest eigenvalue will typically not be zero but close to zero. In this case an eigenvalue cut-off value ϵ ≈ 0, *ϵ *> 0 needs to be specified. An eigenvalue *λ*^1 ^<*ϵ * is considered to be small enough to be treated as zero. Note *λ*^*j *^≥ 0 for all *j *since *H *is positive semi-definite.

The proposed algorithm splits the set of parameter indices {1,..., *n*_*p*_} of a given model into two disjoint subsets which we denote by *I *and *U*. Upon termination, the sets *I *and *U *= {1,..., *n*_*p*_} - *I *contain the indices to the identifiable and the unidentifiable parameters, respectively. We denote the current number of elements in *I *by *n*_*I *_and the current elements in *I *by . The algorithm proceeds as follows.

1. Choose Δ*t*, *t*_*f*_, *ϵ*. Set p* = *p*^lit^. Set *I *= {1,..., *n*_*p*_}, *n*_*I *_= *n*_*p*_, and *U *= ∅.

2. If *I *is empty, stop. The model is not identifiable with respect to any parameter in this case.

3. Fix the parameters *p*_*k*_, *k *∈ *U *to their literature values and consider only the *p*_*k*_, *k *∈ *I *to be variable. Formally, this corresponds to setting *p *to  and treating all remaining parameters *p*_*k*_, *k *∈ *U *as fixed numbers.

4. Calculate *ϕ*(p*) according to equation (9). Calculate the Hessian matrix of *ϕ *with respect to the parameters *p*_*k *_with *k *∈ *I *and evaluate it at *p *= p*. Calculate the eigenvalues *λ*^*j *^and corresponding eigenvectors *u*^*j *^of the Hessian matrix. Assume the eigenvalues to be ordered such that . Assume the eigenvectors to be normalized.

5. If *λ*^1 ^≥ *ϵ *,stop. The model is identifiable with respect to the parameters *p*_*k *_for all *k *∈ *I*.

6. If *λ*^1 ^≥ *ϵ *,select *k *such that . Remove *k *from the set *I*, add *k *to the set *U*, set *n*_*I *_to the current number of elements in *I*, and return to step 2.

The order in which parameters are removed from *I *determines the ranking of parameters from least identifiable to most identifiable.

The method is similar to the approach introduced by Vajda et al. [26]. These authors also analyze the eigenvectors that correspond to small eigenvalues of the Hessian matrix, but they focus on the dependencies between eigenvectors that arise due to special parameter combinations of the form *p*_1_/*p*_2 _or *p*_1_·*p*_2 _in the model. In a two step procedure Vajda et al. first decide which parameters to lump together (for example, *p*_*new *_= *p*_1_·*p*_2_) and subsequently recalculate the eigenvalues and eigenvectors for the system with the new lumped parameters. These two steps are repeated until the smallest eigenvalue is sufficiently large. The lumping step requires manual inspection of the eigenvalues and eigenvectors of the Hessian matrix. While the approach proposed by Vajda et al. worked very well for their example with 5 parameters, it becomes infeasible for models with considerably more parameters. The examples treated in the next section demonstrate that our approach, in contrast, can be applied to models with up to at least a hundred parameters. Moreover, our approach can be carried out automatically, while the approach suggested by Vajda et al. was never intended for this purpose.

#### Correlation method

The correlation method was first introduced by Jacquez and Greif [25] who compared identifiability results achieved with the correlation method to analytical results obtained with a transfer function method (see [14] for a review). Jacquez and Greif [25] consider only small linear compartmental models with up to 3 states and 5 parameters. In most of the examples the correlation and the transfer function method were in agreement.

In the systems biology literature the correlation method was applied by Zak et al. [9] to investigate identifiability of a large genetic regulatory network (nonlinear, 44 states and 97 parameters). More recently, Rodriguez-Fernandez et al. [10] embedded the correlation method into their framework for robust parameter estimation in order to exclude unidentifiable parameters from parameter estimation.

The central idea of the correlation method is to find unidentifiable parameters by investigating the linear dependence of the columns of *S *(for details see Appendix B). The correlation method approximates linear dependence by calculating the sample correlation [35] of two columns  of *S*. The sample correlation is given by

(13)

where

(14)

(15)

(16)

In equations (13)–(16) *corr*(*S*._*i*_, *S*._*j*_), *cov*(*S*._*i*_, *S*._*j*_), *σ *(*S*._*i*_), and  denote the sample correlation between *S*._*i *_and *S*._*j*_, the sample covariance between *S*._*i *_and *S*._*j*_, the sample standard deviation of *S*._*i*_, and the mean of the entries of *S*._*i*_, respectively.

Two linearly dependent columns *S*._*i *_and *S*._*j *_of the sensitivity matrix give rise to an absolute value of the correlation *corr*(*S*._*i*_, *S*_.*j*_) = 1. In the examples treated here, none of the parameters exhibit a correlation of exactly ± 1, however. In the numerical implementation of the method, two columns of *S *are considered correlated if the absolute value of their correlation is equal to or larger than 1 – *ϵ*_*c*_, where *ϵ*_*c *_∈ [0, 1] is a parameter of the algorithm. We stress that while *ϵ*_*c *_is a tuning parameter, the comparison of the identifiability methods is not affected by the choice of *ϵ*_*c*_. As explained below, we systematically vary *ϵ*_*c *_over its entire range 0 ≤ *ϵ*_*c *_≥ 1 in the comparison.

When applying the correlation method to the pathway model examples, two problems arise regularly that have not been discussed by Jacquez and Greif [25]. For one, the method detects pairs of correlated parameters, but there is no criterion which one of the parameters from a pair to consider unidentifiable. Moreover, if more than one pair of correlated parameters is detected, there is no criterion to choose among the pairs.

In order to mitigate these ambiguities we introduce the total correlation

(17)

where

(18)

and *L *denotes the set of indices of those parameters that have not been found to be unidentifiable in any previous iteration. We select the parameter with the highest total correlation for removal. Some cases remain, however, in which two parameters have the same total correlation. In these cases we pick one parameter at random.

In order to be able to compare results to those of the other methods, we use the correlation method to rank model parameters from least identifiable to most identifiable. More precisely, we initially set *ϵ*_*c *_= 0 and *L *= {1... *n*_*p*_} and calculate the total correlations (17) for all parameters in *L*. If parameters with nonzero total correlations exist we select the parameter with highest total correlation to be unidentifiable and remove its index from *L*. If no such parameter exists we increase *ϵ*_*c *_until nonzero total correlations occur. The order in which parameters are removed from *L *creates a ranking of parameters from least to most identifiable. The algorithm is described in detail in Appendix B. Based on the ranking of parameters the method is compared to the other three approaches. The comparison is explained in detail in the subsection entitled Method comparison.

#### Principal component analysis (PCA) based method

Degenring et al. [27] introduce three criteria based on PCA that rank the influence of parameters on model output. Parameters that do not affect the model outputs are unidentifiable by definition and can be removed from the model equations. Degenring et al. successfully use their approach to simplify a complex metabolism model of Escherichia coli K12. A complete description how to get a full ranking using the three criteria can be found in [28]. A pseudo code description of the method can be found in Appendix C.

The PCA based method differs from the other methods described here in that it does not use the sensitivity matrix *S *as defined in equation (7) but a truncated matrix which we refer to by . This matrix  is defined as



where *i *∈ {1,..., *n*_*y*_}. While the sensitivity matrix *S *as defined in equation (7) describes all responses in one matrix,  is created for each response *r*_*i *_separately.

Three PCA based criteria are applied to each of the *n*_*y *_matrices . As a result 3*n*_*y *_parameter rankings are created, which we represent by 3*n*_*y *_lists

(19)

of integers , where *j *∈ {1, 2, 3} and *i *∈ {1,..., *n*_*y*_}. The first ranking position  and the last ranking position  contain the index of the parameter with the lowest and the highest influence on the model output *r*_*i*_, respectively. The lower index *j *∈ {1, 2, 3} denotes the criterion by which the ranking is calculated. We will explain one of the criteria in more detail in order to give the reader an idea of the method. The remaining two criteria are summarized in Appendix C. All 3*n*_*y *_rankings are integrated into one ranking by applying a strategy described in [28], which we briefly summarize below.

Let,  and  denote the *j*th eigenvalue and corresponding eigenvector of , respectively, and assume the eigenvalues to be ordered such that . For each *i *∈ {1,..., *n*_*p*_} evaluate the first criterion as follows.

1. Set *L*_1 _= {1,..., *n*_*y*_}.

2. For *q *from 1 to *n*_*p *_do:

• Find *r*_*l *_such that .

• Set  = *r*_*l*_.

• Set *L*_*q*+1 _= *L*_*q *_- {*r*_*l*_}.

The first criterion loops over the eigenvectors starting with the eigenvector that belongs to the smallest eigenvalue. It identifies the largest absolute entry of each eigenvector and selects the corresponding parameter for removal, if this parameter has not been selected yet. The index of the *k*th parameter selected for removal is ranked at position *k*, where *k *∈ {1,..., *n*_*p*_}.

The remaining two criteria of the PCA based method are similar but differ with respect to the process for chosing *r*_*l*_. For brevity we omit details and refer the reader to Appendix C. A final ranking *J *that incorporates all 3*n*_*y *_previously calculated rankings is obtained as follows. Set *N*_*q *_= ∅, for all *q *= 1,..., *n*_*p*_. Set *J *to an empty list.

For *q *from 1 to *n*_*p *_do:

• For all *j *∈ {1, 2, 3} and all *i *∈ {1,..., *n*_*y*_} set .

• Set .

• Only if *N*_*q *_≠ ∅ append the *N*_*q *_to the list *J*.

First note that an iteration *q *might exist, in which no *N*_*q *_is appended to *J*. Therefore the final number *n*_*J *_of elements in *J *is not necessarily equal to but might be smaller than *n*_*p*_. Further note that entries of *J *do not necessarily correspond to indices of single parameters but to sets of parameter indices. Therefore, a ranking position *J*_*i *_might contain several parameter indices an internal ranking of which cannot be obtained. This ambiguity hampers not only the interpretation of the ranking result but also the comparison with the rankings produced by the other methods. We describe a strategy to deal with the latter problem in section entitled Method comparison.

#### Orthogonal method

The orthogonal method was developed by Yao et al. [24] to analyze parameter identifiability of an Ethylene/Butene copolymerization model with 50 parameters. In the field of systems biology the method has been applied in a framework for model identification [36] and in the identifiability analysis of a NF-*κ*B model [37].

We summarize the concept of the method and refer the reader to Appendix D for details. The method iterates over the columns *S*._*k *_of the sensitivity matrix *S*, with *k *∈ {1,..., *n*_*p*_}. Those columns of *S *that correspond to identifiable parameters are collected in a matrix *X*^*q *^where *q *is the iteration counter. The algorithm starts by selecting the column of *S *with highest sum of squares in the first iteration. In iteration *q *+ 1, *q *columns of *S *have been selected. In the order of their selection these columns form the matrix *X*^*q*^. The next step essentially amounts to selecting the column *S*._*k *_that exhibits the highest independence to the vector space *V *spanned by the columns of *X*^*q*^. More precisely, an orthogonal projection  of *S*._*k *_onto *V *is calculated and  is interpreted as the shortest connection from *V *to *S*._*k*_. The squared length of  is used as measure of independence. If the length of  is near zero, *S*._*k *_is nearly linearly dependent to the columns of *X*^*q*^. Conversely, a large value of  indicates that parameter *p*_*k *_is linearly independent to the columns of *X*^*q*^. In the orthogonal method the parameter *p*_*l *_where  is selected as the next identifiable parameter.

Essentially, the orthogonal method finds those columns of *S *that are as independent as possible. The quantity  can be interpreted as a measure of independence that has been freed from dependencies on previously selected parameters. Therefore not only the influence of a parameter on the outputs, but also the dependencies between the parameters are accounted for by using  as a measure. A visualization of the orthogonal method can be found in Appendix D.

Yao et al. [24] stop the selection process once the length of the maximal  drops below a cut-off value *ϵ*_*o *_≈ 0, the choice of which is rather arbitrary. In the present paper we do not apply this cut-off criterion but merely rank all parameters from most to least identifiable where the terms most identifiable and least identifiable are used in the same sense as in the correlation method. A comparison to the results of the other methods is therefore not affected by the choice of *ϵ*_*o*_. Our approach to comparing the methods is explained in the section entitled Method comparison.

#### Method comparison

The central idea behind our comparison of the methods is the equivalence between the local identifiability of a model and the local positive definiteness of the Hessian matrix (10) at a minimum of the least squares parameter cost function (9) for this model. Due to this theoretical result, which has been established by Grewal and Glover [38], we can test local identifiability at a point in the model parameter space by testing the Hessian matrix (10) for positive definiteness at this point. Since the Hessian matrix is positive definite if and only if all its eigenvalues are strictly positive, we consider a model to be identifiable if the eigenvalues are bounded below by a small strictly positive number *ϵ*. Note the eigenvalue method proposed here is based on the same idea in that it selects those parameters to be unidentifiable that cause eigenvalues smaller than *ϵ*.

By virtue of the relation between local at-a-point identifiability and the eigenvalues of the Hessian matrix we can compare all four identifiability testing methods to one another with the same criterion and an unique parameter value *ϵ*. For a given model we first create a ranking of the parameters from least to most identifiable with each method as described in the previous four sections. For each parameter ranking we then fix the parameter considered to be the least identifiable to its literature value, calculate the Hessian matrix (10) with respect to the remaining parameters, and determine the smallest eigenvalue. In all following steps we additionally fix the next least identifiable parameter and recalculate the Hessian and smallest eigenvalue. This process is carried out until the smallest eigenvalue exceeds *ϵ*. The parameters that need to be fixed in order for the smallest eigenvalue to exceed *ϵ *are considered to be the unidentifiable parameters. Clearly, the smaller the set of unidentifiable parameters, the larger the set of identifiable parameters or, equivalently, the larger the number of parameters for which the least squares parameter estimation problem can be solved. In this sense we consider the method to be the best one that results in the smallest number of unidentifiable parameters in our comparison.

Note in contrast to the other methods, the ranking created with the PCA based method does in general not contain one but several parameters. In such a case we fix all parameters ranked at the same position at once and reevaluate the smallest eigenvalue of the Hessian matrix. The last ranking position  created by the PCA based method contains the indices of all parameters that have not been ranked previously. Fixing of parameters *p*_*k*_, with *k *∈ , would therefore not leave any parameter unfixed. For this reason we consider the PCA method to have failed, if fixing of all parameters with indices in  does not lead to a value of the smallest eigenvalue larger than or equal to *ϵ*.

### Pathway model description

Having introduced the methods to be compared, we briefly summarize the three pathways and the corresponding models used for the identifiability studies.

#### JAK-STAT pathway

The Janus Kinase (JAK) – signal transducer and activator of transcription (STAT) pathway is triggered by cytokines and growth factors. The pathway has an impact on the expression of genes that regulate diverse cellular processes, such as cellular proliferation, differentiation, and apoptosis. A detailed model of the Interferon-induced JAK-STAT pathway is given by Yamada et al. [2]. This model describes the following signal transducing steps. Upon ligand binding the receptor dimerizes thus triggering the activation of receptor-associated JAK. Activated JAK immediately phosphorylates the receptor complex, enabling the binding and subsequent phosphorylation of cytoplasmic STAT. Phosphorylated STAT can dimerize, and finally, dimerized STAT is imported into the nucleus. Here it activates target genes, one of which is the suppressor of cytokine signaling 1 (SOCS1), an important mediator of negative feedback.

#### EGF induced MAP kinase pathway

Mitogen-activated protein (MAP) kinases are involved in mitosis and the regulation of cellular proliferation. The Epidermal Growth Factor (EGF)-induced MAP kinase pathway is centered on a three kinase cascade that terminates with the dual phosphorylation of the third, so-called MAP kinase. EGF stimulation leads to dimerization of the EGF receptor. The dimerized EGF receptor triggers the phosphorylation of Raf, the first kinase of the cascade. Raf, in turn, phosphorylates the mitogen extracellular kinase, MEK, the second kinase in the cascade, which finally phosphorylates the MAP kinase (extracellular signal-related kinase, ERK). Phosphorylated ERK regulates several proteins and nuclear transcription factors and controls the expression of target genes. The model published by Schoeberl et al. [3] additionally includes the internalization of EGF receptor and two further modules: an SH2-domain containing protein (SHC) dependent module and an SHC independent one.

#### NF-*κ*B pathway

Nuclear Factor-*κ*B (NF-*κ*B) is a transcription factor, which regulates genes involved in inflammation, immune response, cell proliferation, and apoptosis. In the unstimulated cell NF-*κ*B is captured in the cytoplasm by the protein inhibitor of NF-*κ*B (I*κ*B*α*). Upon stimulation by pathway activating signals such as the Tumor Necrosis Factor (TNF), the I*κ*B kinase (IKK) is activated. Activated IKK phosphorylates I*κ*B*α *thus inducing its degradation. As a consequence NF-*κ*B is released, translocates into the nucleus and regulates effector genes. NF-*κ*B induces its own downregulation by inducing the production of two proteins: 1) I*κ*B*α *that inhibits NF-*κ*B by relocating it to the cytoplasm and 2) the zink-finger protein A20 that represses IKK and consequently indirectly inhibits NF-*κ*B.

## Results

In this section the results of the identifiability analysis are reported. All simulations are carried out with literature values *p*^lit ^for the model parameters, which are taken from the original publications of the models [1-3]. We assume that all state variables are available as outputs, i.e. *y*(*t*) = *x*(*t*) and *n*_*y *_= *n*_*x*_. Clearly, it is far from realistic to assume all state variables could be measured in an actual experiment for any of the signaling pathways. Since even under this strong assumption the models turn out not to be identifiable, however, it is not reasonable to investigate the identifiability with fewer outputs. In fact our identifiability results show that the models are overparameterized even under the very strong assumption of all state variables being measurable. The algorithmic parameter *ϵ *is set to *ϵ *= 10^-4^. While this value is motivated by our experience with numerical parameter estimation problems, we admit that it is somewhat arbitrary. Note, however, the comparison of the identifiability testing methods is consistent in that the same value *ϵ *= 10^-4 ^is used throughout. Output values *y*(*t*) are recorded from the simulations at equidistant points in time. The JAK-STAT model is simulated for 28800 seconds (8 hours) and the response function is recorded with a time step of Δ*t *= 360 *s*. The corresponding values for the MAP kinase and NF-*κ*B models are 3600 s (1 hour), Δ*t *= 120 *s*, and 21600 s (6 hours), Δ*t *= 360 *s*, respectively. These values are not critical. Similar results are obtained with other values of *t*_*f *_and Δ*t *(data not shown).

### JAK-STAT pathway analysis

**Figure 1:**
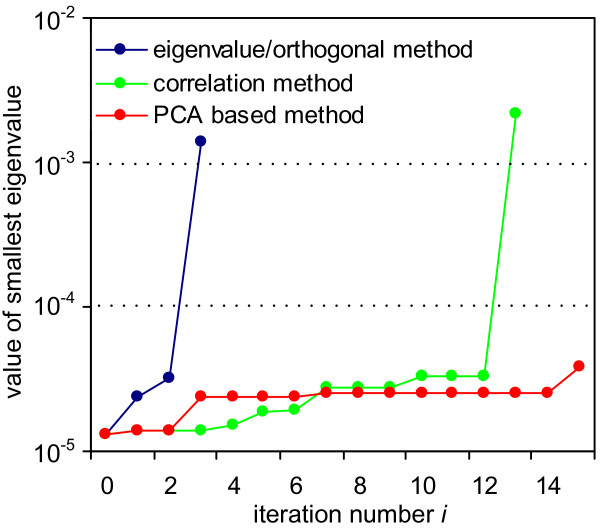
**Results of the methods applied to the JAK-STAT model**. The graph illustrates how the value of the smallest eigenvalue changes, when one parameter is fixed in each iteration. The y-axis shows the value of the smallest eigenvalue of the Hessian matrix *H*. Parameters are fixed until the value of the smallest eigenvalue is larger than *ϵ *= 10^-4^.

Figure 1 summarizes the results of the analysis of the JAK-STAT model. The figure shows the value of the smallest eigenvalue as a function of the number of fixed parameters. Before discussing the parameters that are considered to be unidentifiable by the various methods in detail, some results can already be inferred from Figure 1. In iteration *i *= 0, i.e. before any parameter is fixed in the model, the smallest eigenvalue *λ*^1 ^equals 1.3·10^-5^. This implies the model is not identifiable even if all state variables *x*(*t*) are assumed to be available. The smallest eigenvalue exceeds *ϵ *= 10^-4 ^after the first three parameters regarded as unidentifiable by the eigenvalue and the orthogonal method are fixed (specifically, kb7, kb30, and kb5). In contrast, the first 13 parameters ranked by the correlation method need to be fixed to obtain a smallest eigenvalue larger than the threshold *ϵ *= 10^-4^. Since the eigenvalue and orthogonal method find an identifiable model by fixing a much smaller subset of the parameters, we conclude these methods outperform the correlation method in this particular case. The PCA based method fails for this example.

Some of the results generated by the four methods are worth inspecting more closely. In the first and second iteration of the eigenvalue method there exists more than one dominant contribution to the degenerate direction, cf. Table 1. In the first iteration there are two co-dominant entries (last column in Table 1). The corresponding parameters are kb5 and kb30. In the second iteration there exists one co-dominant entry, which corresponds to the parameter kb5. In the last iteration no co-dominant entries exist and the parameter kb5 is solely responsible for the degenerate direction. It turns out that selecting the parameter corresponding to the maximum entry of |*u*^1^| as done here is sufficient to find a minimal set of identifiable parameters. Interestingly, we can see in this example that after three iterations our method finds the parameters corresponding to the co-dominant entry of all previous iterations.

**Table 1:** Results of the eigenvalue method for the JAK-STAT model

iteration	removed parameter	max. entry of |*u*^1^|	co-dominant entries of |*u*^1^|
1	kb7	0.725	0.56, 0.4

2	kb30	0.728	0.68

3	kb5	0.997	-

The correlation method indicates that 13 parameters need to be fixed in order for the model to be locally identifiable (Figure 1). The sets of parameters removed by the eigenvalue and orthogonal method have only kb7 in common with the parameters selected by the correlation method. In the correlation method, the removal of parameter kb7 occurs in the last iteration and results in a clear increase of the smallest eigenvalue by a factor of about 100 (from 3.3·10^-5 ^to 2.2·10^-3^, see Figure 1). Surprisingly, previous iterations of the correlation method hardly have an effect on the minimum eigenvalue.

Table 2 shows ranking *J *as produced by the PCA based method for the JAK-STAT model. Each ranking position *J*_*i *_corresponds to a set of parameter indices, the size of which is shown in the table. The major problem of the PCA based method becomes apparent here. Ranking *J *has 5 positions. Fixing the 15 parameters of the first four positions of *J *is not sufficient to get an identifiable model. Fixing the set of 36 parameters at the last ranking position would not leave any parameter unfixed. Therefore the method has failed in finding a set of identifiable parameters. The ambiguity of the method is apparent from positions four and five of the ranking. The sets *J*_4 _and *J*_5 _contain 8 and 36 parameters, respectively, which cannot be ranked internally by the PCA based method. It is interesting to note that the first ranking position of *J *is not filled until iteration 45 (data not shown), six iterations before the last iteration. No nonempty intersection of the first 44 positions of all rankings  with *j *∈ {1, 2, 3} and *i *∈ {1,..., *n*_*y*_} exists. This indicates that large parts of the three different rankings , *j *= 1, 2, 3 do not agree with respect to the order of parameters.

**Table 2:** Ranking created by the PCA based method for the JAK-STAT model

ranking position *i*	size of *J*_*i*_
1	1

2	2

3	4

4	8

5	36

**Figure 2:**
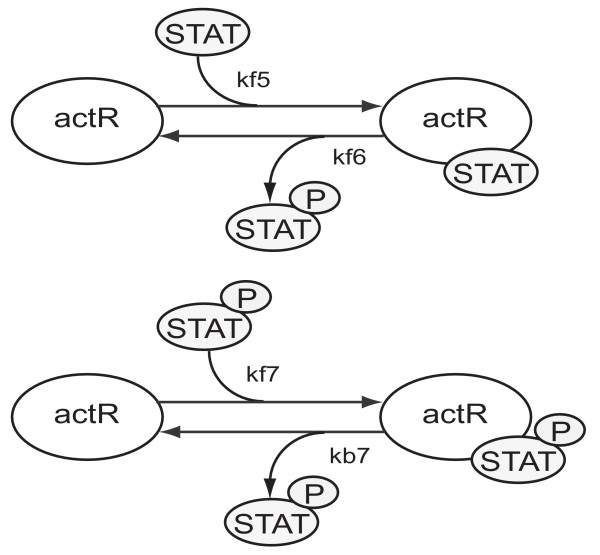
**Part of the JAK-STAT model to be simplified**. The activated receptor is denoted by actR. The encircled P indicates that STAT is phosphorylated.

The parameters removed by the eigenvalue and orthogonal methods point the way to possible simplifications of the pathway models. The parameter kb7 describes the dissociation of phosphorylated STAT from the activated receptor complex. This reaction, however, is not the dissociation that immediately follows the phosphorylation of STAT by the activated receptor (Figure 2, kf6). Apart from the association of unphosphorylated STAT to the receptor (Figure 2, kf5), the phosphorylation of STAT by the activated receptor and the subsequent dissociation of phosphorylated STAT from the activated receptor (Figure 2, kf6), the model also permits the reassociation of already phosphorylated STAT from the cytoplasm to the activated receptor (Figure 2, kf7). The dissociation described by parameter kb7 is the reverse reaction to the reassociation described by kf7. We claim these steps are less important, since the key event is the phosphorylation of cytoplasmic STAT by the activated receptor. Removing kb7 alone creates a sink for phosphorylated STAT and activated receptor, since the dissociation of both species does not exist anymore. Therefore the model can only be simplified by removing both the reaction kb7 is involved in and the reaction kf7 is involved in. The main effects of these reactions are 1) to reduce the amount of free phosphorylated STAT and 2) to reduce the amount of free activated receptor. After removing both reactions, effect 1) can be imitated by appropriately decreasing those rate constants involved in reactions with free phosphorylated STAT. Effect 2) can be compensated by appropriately decreasing those rate constants that are involved in reactions with free activated receptor.

The second fixed parameter, kb30, corresponds to the rate of dissociation of STAT from the complex formed by SOCS1 and the activated receptor complex. In order to get a simplified model that is identifiable, we may remove this step. This is justified, because the key function of SOCS1 is not its binding to STAT, but its inhibition of the activated receptor complex [39]. The latter is still ensured in the simplified model.

The third fixed parameter, kb5, describes the dissociation of receptor-bound unphosphorylated STAT. Assuming that STAT binds irreversibly to the activated receptor, the model can be simplified. In this scenario, STAT dissociates from the receptor only after STAT phosphorylation has occurred. Due to the presence of PPX, which can dephosphorylate cytoplasmic STAT, this change to the model need not result in an increase of the concentration of phosphorylated STAT.

We stress that the fact that kb7, kb30, and kb5 render the model unidentifiable does not imply the corresponding reactions do not exist. Our results imply, in contrast, that the model is too complex in the sense that these parameters cannot be determined by model based parameter estimation. Note that this result holds true even if we assume data for all the state variables to be available.

### Map Kinase pathway analysis

Before any parameter of the MAP kinase model is fixed, the smallest eigenvalue of the Hessian matrix (10) equals 3.3·10^-13^. This implies the model is not identifiable. The eigenvalue and the orthogonal method again outperform the correlation method in that they find a significantly smaller set of parameters to be responsible for the unidentifiability of the model. The PCA based method does not lead to an identifiable method.

In the eigenvalue method the maximal components of the respective eigenvectors are near 1 (data not shown) in both iterations. As a result, two parameters, k15 and k1, can unambiguously be fixed in the first and second iteration, respectively.

**Figure 3:**
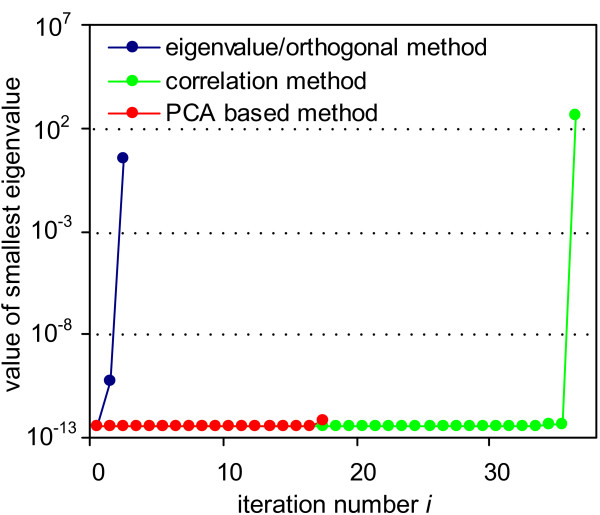
**Results of the methods applied to the MAP kinase model**. Until iteration number 17 the green curve lies below the red curve.

The correlation method needs to fix 36 parameters to arrive at an identifiable model. Fixing the first 35 parameters does not significantly increase the smallest eigenvalue (Figure 3). Only when the last parameter is fixed a dramatic increase of the smallest eigenvalue from about 4.0·10^-13 ^to 4.3·10^2 ^results. The parameter k15 is also removed by the eigenvalue and the orthogonal method, which indicates the importance of k15 for identifiability. The other parameter removed by the eigenvalue method, k1, is selected in the 2nd iteration of the correlation method. Therefore, the set of 36 parameters found by the correlation method is a superset of the 2 parameters selected by the eigenvalue and the orthogonal method. Closer inspection of the first iteration in the correlation method reveals a high maximal total correlation, , of 7.98. This high value is caused by couplings of the selected parameter with eight other parameters. Despite this high total correlation value of 7.98, the removal of the corresponding parameter does not cause a significant increase of the smallest eigenvalue. Just as in the previous example, removing parameters with high correlations does not necessarily improve identifiability.

The ranking found by the PCA based method has five positions (Table 3). When the 17 parameters that correspond to *J*_1_, *J*_2_, *J*_3_, and *J*_4 _are fixed, the model remains unidentifiable. Fixing the parameters that correspond to *J*_5 _is not reasonable, since doing so would not leave any parameters unfixed. Due to this result, we consider the PCA based method to have failed.

**Table 3:** Ranking created by the PCA based method for the MAP kinase model

ranking position *i*	size of *J*_*i*_
1	1

2	1

3	3

4	12

5	78

In summary, the eigenvalue method and the orthogonal method identify the smallest set of parameters that needs to be fixed in order to create an identifiable model. The first parameter, k15, is involved in internalization processes represented by more than twenty reactions [3]. Comprehensive changes to this part of the model that are necessary to ensure identifiability cannot be addressed here. The second parameter, k1, describes the binding of EGF to its receptor. This essential step cannot be simplified. However, experimental data on this reaction exists in the literature [40,41]. Our analysis suggests using this kind of independent information to fix the value of k1 before attempting to determine the remaining parameters by parameter estimation.

### NF-*κ*B pathway analysis

**Figure 4:**
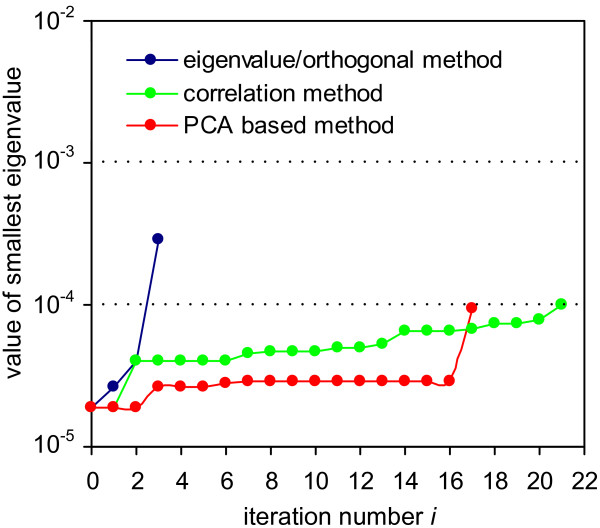
**Results of the methods applied to the NF-*κ*B model**.

For the NF-*κ*B pathway model, the smallest eigenvalue *λ*^1 ^= 1.9·10^-5 ^results if no parameters are fixed (Figure 4). This indicates that the model is not identifiable for the published parameter values [1]. The eigenvalue and the orthogonal method again find the same 3 unidentifiable parameters, c4a, c4, and t1. The correlation method selects a considerably larger set of 21 parameters. The PCA based method fails.

In the first three iterations of the eigenvalue method, the largest components of *u*^1 ^have absolute values close to one (data not shown). The method therefore selects these parameters unambiguously.

The correlation method needs to fix 21 parameters until the smallest eigenvalue is greater than *ϵ*. The last parameter, the fixing of which leads to an identifiable model (Table 4), has been selected with a value of *ϵ*_*c *_= 0.55. This is not reasonable anymore, since two columns of *S *with a correlation of 1 - *ϵ*_*c *_= 0.45 can hardly be considered correlated. Therefore we conclude that the correlation method fails for this example.

**Table 4:** Results obtained with the correlation method for the NF-*κ*B model

rank	removed parameter*	||·10^-05^	
1	c1(0.99)	1.90	1.000
2	c1a(0.99)	3.98	1.000
3	c3(0.99)	3.99	0.998
4	a3(0.97)	4.06	1.958
5	c3a(0.96)	4.07	0.967
6	c3c(0.93)	4.07	1.874
7	c4a(0.93)	4.61	0.938
8	c4(0.92)	4.62	0.930
9	c1c(0.92)	4.62	0.927
10	kdeg(0.84)	4.65	0.849
11	c2a(0.81)	4.96	0.819
12	c2(0.80)	4.96	0.805
13	c5a(0.74)	5.34	0.743
14	a2(0.72)	6.54	0.727
15	a1(0.64)	6.64	0.643
16	i1(0.56)	6.64	0.565
17	c6a(0.50)	6.82	0.506
18	k2(0.50)	7.31	0.503
19	e2a(0.46)	7.42	0.470
20	e1a(0.45)	7.79	0.459
21	kv(0.45)	10.08	0.455

* The value of 1 – *ϵ*_*c *_is given in brackets. See the text for a discussion.

The PCA based method creates a ranking *J *with six positions (Table 5). Fixing the parameters that correspond to *J*_1 _to *J*_5 _does not result in an identifiable model. Fixing the parameters corresponding to *J*_6 _will not leave any parameters unfixed. Therefore the method has failed in producing an identifiable model. After fixing all parameters from *J*_1 _to *J*_5 _the resulting smallest eigenvalue *λ*^1 ^= 9.5·10^-5 ^is close to *ϵ*. Having a look at the parameters in the ranking *J *we can see that *J*_1 _contains parameter t1, and *J*_5 _contains parameter c4. Both parameters are also found by the eigenvalue and the orthogonal method.

**Table 5:** Ranking created by the PCA based method for the NF-*κ*B model

ranking position *i*	size of *J*_*i*_
1	1

2	2

3	3

4	1

5	10

6	12

The three parameters selected for removal by the eigenvalue and the orthogonal method indicate how to simplify the model. The first two parameters, c4a and c4, describe the only two translation steps in the model: the translation of I*κ*B*α *mRNA and A20 mRNA, respectively. By lumping together transcription and translation into one protein synthesis step the two unidentifiable parameters c4a and c4 can be removed from the model. The third parameter selected by the eigenvalue and orthogonal method, t1, describes the dissociation of activated IKK from I*κα*. Since this reaction is a key step of the model the only option for simplifying this part of the model is to remove IKK entirely. As a consequence A20 becomes obsolete, since the only function of A20 is the regulation of IKK. If we also remove A20, the resulting simplified model closely resembles the minimal model proposed by Krishna et al. [42].

## Discussion

The eigenvalue, orthogonal, and correlation methods find the desired subset of identifiable parameters and the desired subset of unidentifiable parameters in all cases. The eigenvalue and orthogonal methods give the same results for each of the models. The subsets of unidentifiable parameters that result from the correlation method are larger than those that result from the eigenvalue or orthogonal method in all cases. The PCA based method fails for all three examples.

The four identifiability testing methods use different approaches to finding unidentifiable parameters. In order to carry out a meaningful comparison of the methods, we need a single identifiability criterion that can be applied to all methods. The positive definiteness of the Hessian matrix (10) of the least squares parameter estimation problem (9) is an appropriate and concise criterion for this purpose. Ultimately, the use of this criterion is justified by the equivalence between local at-a-point identifiability and the positive definiteness of the Hessian matrix (10). This equivalence was established by Grewal and Glower [38].

Since the Hessian matrix is positive definite if and only if all its eigenvalues are strictly positive, we consider a model to be identifiable if the eigenvalues of the Hessian matrix (10) are bounded below by a small strictly positive number *ϵ*. Based on experience with parameter estimation problems we set *ϵ *= 10^-4^. While this choice is arguably arbitrary, the comparisons of the methods are meaningful since the same value of *ϵ *is applied in all comparisons.

**Figure 5:**
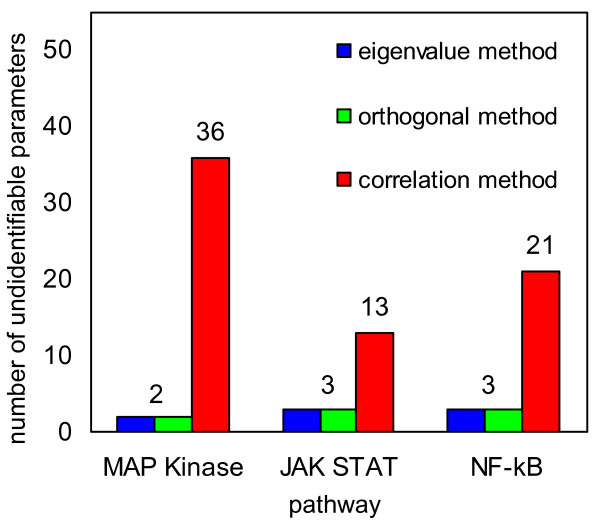
**Summary of the method comparison**. Since the PCA based method fails in all three cases, it is not included here.

The numbers of unidentifiable parameters are summarized in Figure 5 for the four methods and the three models. More precisely, the Figure shows how many parameters are selected by each method to be fixed in order to obtain a Hessian matrix with all eigenvalues larger than *ϵ *= 10^-4^. From Figure 5 it is apparent that fewer parameters need to be fixed than those selected by the correlation method to obtain a positive definite Hessian matrix or, equivalently, an identifiable model in all examples. In this sense we conclude that the eigenvalue and orthogonal method outperform the correlation method. Since the PCA based method fails in all three examples, we consider it to be inferior to the other methods.

In the remainder of the section we discuss the differences between the methods.

### Eigenvalue method vs. orthogonal method

The eigenvalue method can be motivated in two different ways. For one, it can be interpreted as a convexity analysis of the minimization of the least squares cost function *ϕ *(9) at a nominal point p*, in parameter space. If *ϕ *is too flat at p*, the method identifies the parameters that cause this flatness. Secondly, the method can be looked upon as an approach to identifying linearly dependent columns of *S*. If *S*^*T *^*S *has zero eigenvalues it is not invertible, which implies that *S *has linearly dependent columns. By fixing those parameters that cause *S*^*T *^*S *to be not invertible, the linear independence of columns of *S *can be ensured. The orthogonal method selects those columns of *S *that have the largest possible orthogonal distance to previously selected columns. This way linearly independent columns are found. Essentially, the eigenvalue and orthogonal method use the same criterion, but the eigenvalue methods discards those parameters that render a model unidentifiable while the orthogonal method selects the identifiable parameters. Both methods give the same results for the examples treated here.

A formal analysis of the two methods reveals the computational complexity to depend on the magnitudes of *n*_*y *_*n*_*t *_and *n*_*p *_(see Appendix E). If , as is the case in the JAK-STAT and the NF-*κ*B example, the eigenvalue method is an order of magnitude in the number of parameters faster than the orthogonal method. If we assume , as is the case for the MAP kinase example, the eigenvalue method is slightly faster than the orthogonal method. If *n*_*y *_*n*_*t *_≈ *n*_*p*_, both methods have the same computational complexity. We stress the complexity analysis is carried out under assumptions that are unfavorable for the eigenvalue method in that the eigenvalue method is assumed to require a full ranking to separate the identifiable from the unidentifiable parameters. In the three examples treated here this is clearly not the case, however. In fact the eigenvalue method stops after three, two, and three iterations in the JAK-STAT, the MAP kinase, and the NF-*κ*B case, respectively, and therefore turns out to be far less computationally expensive than under the worst case assumption used in Appendix E.

### Technical drawbacks of the correlation method

From a technical point of view, the correlation method suffers from two drawbacks. These drawbacks cause the differences between the number of unidentifiable parameters found by the correlation method as compared to the orthogonal and eigenvalue method. First, the correlation *corr*(*S*._*i*_, *S*._*j*_) is only an approximation of the linear dependency of columns of *S*. While two columns that are linearly dependent result in a correlation of ± 1, columns that have a correlation of ± 1 need not necessarily be linearly dependent (see Appendix F for an example). Therefore, the correlation method may find false positive unidentifiable parameters. Secondly, the correlation *corr*(*S*._*i*_, *S*._*j*_) is only a pair wise measure for any two columns *S*._*i *_and *S*._*j *_of *S*. Linear dependence of a set of more than two columns, which does not contain a linearly dependent pair, is not detected.

### Technical drawbacks of the PCA based method

The PCA based method differs from the three other methods in several aspects. The PCA based method does not use the sensitivity matrix *S*, but a sensitivity matrix is calculated for each component *r*_*i *_of the response function separately. In contrast to the other methods, couplings between the components *r*_*i *_are therefore not considered. A second important difference is that the PCA based method does not use a single criterion, but a combination of criteria which all must hold for a parameter to be considered unidentifiable. Due to this combination of criteria, the method does not select a single parameter in each iteration but a set of parameters. Finally, some parameters that cause *S *not to have full rank are not found by the PCA based method in all three examples. For this reason, we consider the PCA based method to be inappropriate for the identifiability testing of the three treated pathways.

## Conclusion

We proposed a new approach to finding identifiable and unidentifiable parameters of large mechanistic models. The method, which we refer to as the eigenvalue method, was applied to existing models of the JAK-STAT, the MAP kinase, and the NF-*κ*B pathways. All three pathway models turned out not to be identifiable. In fact our results show that the models would not be identifiable even if all state variables could be measured precisely at a high frequency. The models must therefore be considered to be severely overparameterized. Note state identifiability investigated here is a necessary condition for output identifiability. Since the models turn out not even to be state identifiable, we refrained from further investigations of output identifiability.

The identifiability analysis reveals how to simplify the models in order to arrive at identifiable systems. For the JAK-STAT pathway an identifiable model can be obtained by simplifying only three reactions. For the NF-*κ*B pathway model the results lead to a simplified model that closely resembles the minimal model published by Krishna et al. [42].

We presented a detailed comparison of the proposed eigenvalue approach to three established methods for identifiability testing, namely, the correlation method [25], the PCA based method [27], and the orthogonal method [24]. These three methods and the new approach suggested are of general interest, because they can be applied to a broad class of nonlinear systems of ordinary differential and algebraic equations. Essentially, the methods were compared with respect to their ability to find an identifiable subset of model parameters for each of the three pathway models. Despite algorithmically different, the eigenvalue and the orthogonal method result in the same subsets of identifiable parameters for each of the examples. Moreover, these two methods outperform the other methods in that they find the largest subsets of identifiable parameters.

## Authors' contributions

TQ and MM developed the eigenvalue method. The discussed method were implemented and compared by TQ. TQ and MM wrote the article.

## Appendix

### A: Example with co-dominant parameters

We present an illustrative example to motivate the notion of co-dominant parameters. Consider the trivial system  = (*p*_1 _+ *p*_2_)·*x*_1 _with *y*_1 _= *x*_1_. Using the notation introduced below equation (10), the eigenvalue *λ*^1 ^equals 0 (independent of the choice of *p*_1_, *p*_2_, sampling times, and initial condition) with |*u*^1^| = (0.707, 0.707)^*T*^. From the simple model equation it is apparent that *p*_1 _and *p*_2 _are not identifiable separately, but only their sum *p*_1 _+ *p*_2 _can be estimated from data. As a result, *p*_1 _and *p*_2 _are co-dominant as indicated by the result . Fixing either one of the parameters renders the other one identifiable.

### B: Correlation method

We first summarize theoretical aspects of the correlation method and subsequently give a detailed description of the algorithm.

#### Theory

Let Δ*p *= *p *- p* describe the deviation of a parameter vector *p *from the true parameter vector p*. For sufficiently small ||Δ*p*||_2 _the response function (4) can be approximated by its linearization at p*:



for all *i *∈ {1,..., *n*_*y*_} and *j *∈ {1,..., *n*_*t*_}. The sum of squared errors of the linearized response functions and the measured outputs *y*_*i *_(*t*_*j*_) reads

(20)

The last equality holds, because the *y*_*i *_(*t*_*j*_) are assumed to be noise free measurements and p* are the true parameter values by assumption. Therefore *r*_*i*_(*t*_*j*_, p*, *x*_0_) - *y*_i_(*t*_j_) = 0 for all *i *∈ {1,..., *n*_*y*_} and *j *∈ {1,..., *n*_*t*_}.

Let  denote the least squares estimates of Δ*p*, i.e. the particular values of Δ*p *that minimize equation (20). The necessary condition for optimality  is equivalent to the so called normal equation *S*^*T *^*S *Δ*p *= 0. Solving the normal equation for Δ*p *therefore results in the least squares estimate .

If *S*^*T *^*S *has full rank, the normal equation only admits the solution Δ*p *= 0. This implies Δ*p *= 0, or equivalently  = p*, is the unique minimizer of the least squares function *R*(Δ*p*). Consequently, the model parameters *p *are locally identifiable by definition. Note only local identifiability can be inferred, since an approximation by linearization was introduced. If, on the other hand, *S*^*T *^*S *does not have full rank, there exists a non-trivial solution  ≠ 0 of the normal equation. As a consequence, a parameter vector  ≠ p* exists that results in the same model response as p*. Therefore the model parameters *p *are not identifiable by definition.

The matrix *S*^*T *^*S *has full rank if and only if the columns of *S *are linearly dependent [43]. The central idea of the correlation method is to detect linear dependence of two columns of *S *by calculating the correlation of these columns. Parameters corresponding to columns of *S *with a correlation of ± 1 are considered unidentifiable.

#### Algorithm for the correlation method

Let *C *denote the matrix that contains those absolute correlation values between columns of *S *that exceed the threshold 1 - *ϵ*_*c*_. *C *is given by:



where *S*._*j *_denotes the *j*th column of *S *and corr* (*S*._*i*_, *S*._*j*_) is defined in equation (18). The algorithm proceeds in the following way:

1. Choose Δ*t *and an initial guess p*. Set *ϵ*_*c *_= 0, *I *= {1,..., *n*_*t*_}, and initialize *U *to an empty list. Calculate the sensitivity matrix *S*.

2. Calculate the matrix C.

3. For all *i *∈ *I *calculate (*I*) as defined in equation (17).

4. If *I *is empty, stop. The list *U *contains the parameter indices in the order of the least to most identifiable parameter.

5. Find *r*_*q *_such that .

6. If  = 0, increase *ϵ*_*c *_by 0.01 and return to step 2. Otherwise, remove *r*_*q *_from *I*, append *r*_*q *_to the list *U*, and return to step 3.

The list *U *contains the desired result as explained in step 4. Note *ϵ*_*c *_is incremented in steps of Δ*ϵ*_*c *_= 0.01. The choice of this increment is not critical as long as it is small enough. If Δ*ϵ*_*c *_is chosen smaller than necessary the same ranking of parameters will be obtained but the algorithm may require more computation time.

### C: PCA based method

We explain the second and third PCA based criterion and present the algorithm for the PCA based method. The first criterion is explained in the section entitled Methods.

#### Description of the second and third criterion of the PCA based method

Let  and  denote the *j*th eigenvalue and corresponding eigenvector of , respectively, and assume the eigenvalues to be ordered such that . For each *i *∈ {1,..., *n*_*p*_} evaluate the second and third criterion as follows.

• Second criterion:

Set *L*_1 _= {1,..., *n*_*y*_}.

For *q *from 1 to *n*_*p *_do:

- Find *r*_*l *_such that .

- Set  = *r*_*l*_.

- Set *L*_*q*+1 _= *L*_*q *_- {*r*_*l*_}.

The second criterion is based on the sum of squared eigenvector entries

(21)

Note in this sum, *i *and *j *are fixed, therefore the sum is not carried out over components of a particular eigenvector, but the *l*th entries of all eigenvectors are summed. The parameter with the largest value of (21) is selected.

• Third criterion:

Set *L*_1 _= {1,..., *n*_*y*_}.

For *q *from 1 to *n*_*p *_do:

- Find *r*_*l *_such that .

- Set .

- Set *L*_*q*+1 _= *L*_*q *_- {*r*_*l*_}.

The third criterion loops over the eigenvectors but, in contrast to the first and second criterion, starts with the eigenvector that belongs to the largest eigenvalue. As in the first criterion, parameters are selected based on the absolute value of the eigenvector component. The index of the parameter selected first will be placed last in ranking , the index of the parameter selected second will be placed at position *n*_*p *_- 1, and so on.

#### Algorithm for the PCA based method

1. Choose Δ*t *and an initial guess p*. For all *i *∈ {1,..., *n*_*y*_} let , , and  be empty lists. For all *q *∈ {1,..., *n*_*p*_} set *N*_*q *_= ∅. Set *J *to an empty list.

2. For all *i *∈ {1,..., *n*_*y*_} calculate , its eigenvectors , and the corresponding eigenvalues , *j *∈ {1,..., *n*_*p*_}.

3. For *i *∈ {1,..., *n*_*y*_} calculate , , and  by applying ranking criterion one to three as described in the Method section. Let  denote the *k*th entry of .

4. For *q *from 1 to *n*_*p *_do:

• For *j *∈ {1, 2, 3} and *i *∈ {1,..., *n*_*y*_} set .



• Only if *N*_*q *_≠ ∅ append *N*_*q *_to the list *J*.

The final ranking that combines the PCA results of all  and all three criteria is given by *J*, where the first position in *J *corresponds to the least and the last position in *J *corresponds to the most identifiable parameter.

### D: Orthogonal method

In this part we present a geometric interpretation the orthogonal method, followed by a description of algorithmic details.

### Visualization of the concept of the orthogonal method

**Figure 6:**
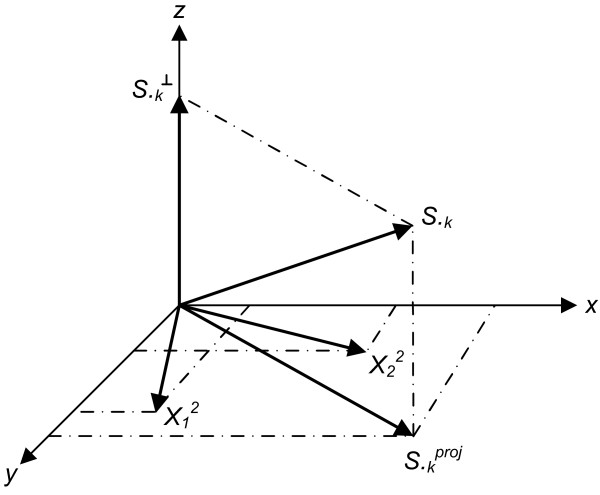
**Visualization of the concepts of the orthogonal method**.

The orthogonal method is visualized in Figure 6 for *q *= 3. Two columns of *S *have already been selected and represent the first and the second column of *X*^2 ^denoted by  and , respectively. These two columns span the vector space *V*_2_. Without restriction this vector space is assumed to be the *xy *plane and *k *is the next parameter to be chosen.

### Algorithm for the orthogonal method

Algorithmic details for the orthogonal method are given below.

1. Choose Δ*t *and an initial guess p*. Initialize  to a list of zeros.

2. Calculate the sensitivity matrix *S*.

3. Find *r*_1 _such that , set , and *L*_1 _= {1,..., *n*_*p*_} - {*r*_1_}.

4. For *q *from 1 to *n*_*p *_do:

• Calculate *P*_*q *_as given by equation (22).

• Find *r*_*q *_such that , with  corresponding to the *k*th column of *P*_*q*_.

• Set  ≥ *ϵ*_*o*_:

set ,

set ,

and set *L*_*q*+1 _= *L*_*q *_- {*r*_*q*_}.

The ranking is given by *W*^*ortho*^. The shown implementation ignores the stopping criterion applied in the original literature, to ensure a complete ranking.

### E: Computational complexity of the orthogonal and the eigenvalue method

The numerical operations of the two methods that dominate the number of necessary numerical operations are 1) matrix inversions and eigenvalue/eigenvector calculations, which both require *O*(*N*^3^) operations for *N *× *N *matrices, and 2) matrix multiplications, which require *N*·*M*·*L *operations for the multiplication of an *N *× *M *by an *M *× *L *matrix.

In what follows, we assume the eigenvalue method is used to rank all model parameters. This overestimates the computational cost of the eigenvalue method, since the method stops once an identifiable set of parameters has been found.

#### Computational complexity of the orthogonal method

In compact notation the orthogonal projection in iteration *i*, where *i *∈ {1,..., *n*_*p*_} is arbitrary but fixed, can be written as:

(22)

The major runtime costs result from the computation 1) of *Y *and 2) of *Y S' *in equation (22). The number of operations necessary to calculate *Y *is:

(23)

(24)

where all iterations *i *= 1,..., *n*_*p *_have been accounted for. The first and second term in the sum of equation (23) account for the multiplication of *X*^*i *^by (*X*^*iT *^*X*^*i*^)^-1 ^(*n*_*y *_*n*_*t *_× *i *matrix times *i *× *i *matrix) and the inversion of the matrix *X*^*iT *^*X*^*i *^(*i *× *i *matrix), respectively. The third term accounts for the cost of multiplying *X*^*iT *^by *X*^*i *^(*i *× *n*_*y *_*n*_*t *_matrix times *n*_*y *_*n*_*t *_× *i*). This term reduces from *i*^2 ^*n*_*y *_*n*_*t *_to *in*_*y *_*n*_*t*_, the cost or calculating the last row of *X*^*iT *^*X*^*i*^, since *X*^*i*-1*T *^*X*^*i*-1 ^is available from the previous iteration:



Here  denotes the *i*th column of *X*^*i*^. The last row is equal to the last column, since *X*^*iT *^*X*^*i *^is symmetric. The cost for *i *vector multiplications  with 1 ≤ *k *≤ *i *amounts to *in*_*y *_*n*_*t*_, which gives rise to the last term in the sum in equation (23).

The number of operations necessary to calculate *Y S' *is:

(25)

The first term in the sum accounts for the multiplication of *Y *by *S' *(*n*_*y *_*n*_*t *_× *i *matrix times *i *× *n*_*p *_matrix). The second term accounts for the calculation of *S' *from the product *X*^*iT *^*S *(*i *× *n*_*y *_*n*_*t *_matrix times *n*_*y *_*n*_*t *_× *n*_*p *_matrix). The latter reduces to the cost *n*_*y *_*n*_*t *_*n*_*p *_for calculating the last row of *S'*, since *X*^*i*-1*T *^*S *is vailable from the previous iteration:



where  denotes the last column of *X*^*i *^and *S*._*i *_denotes the *i*th column of *S*.

The subtraction of *S *from *Y S' *(*n*_*y *_*n*_*t *_× *n*_*p *_matrix minus *n*_*y *_*n*_*t *_× *n*_*p *_matrix) in equation (22) requires *O*(*n*_*y *_*n*_*t *_*n*_*p*_) operations and therefore can be neglected.

In summary the dominant parts in equation (24) and (25) result in a computational complexity of order .

#### Computational complexity of the eigenvalue method

For ease of presentation the description of the algorithm in the Methods section slightly differs from the actual implementation. In the implemented version the Hessian matrix *H *= *S*^*T *^*S *has to be calculated only once at the beginning of the algorithm, which requires  operations (multiplication of a *n*_*p *_× *n*_*y *_*n*_*t *_matrix with a *n*_*y *_*n*_*t *_× *n*_*p *_matrix). In each iteration *i*, the current Hessian *H*^*i *^can be obtained by removing the rows and columns from *H *that correspond to fixed parameters. In each iteration the eigenvalues of the current Hessian matrix have to be calculated. The number of operations necessary for the eigenvalue method is as follows.

(26)

(27)

The first term accounts for the initial calculation of the Hessian matrix. The second term accounts for the eigenvalue calculation.

#### Comparing computational complexity of the eigenvalue and the orthogonal method

The computational complexity of the eigenvalue method is of order  (see equation (27)), whereas the computational complexity of the orthogonal method is of order  (see equation (24)). For a comparison we have to distinguish between the following three cases. In the first case we assume *n*_*y *_*n*_*t *_≤ *n*_*p*_. In this case the complexity of both methods is dominated by the term  and both method are equally efficient. In the examples treated here, this case does not occur. In the second case we assume . Here the computational complexity of the eigenvalue and the orthogonal method reduce to  and , respectively. This case applies to the MAP-kinase example and the eigenvalue method is faster than the orthogonal method. The difference is the more pronounced the larger *n*_*y *_*n*_*t *_- *n*_*p*_. In the last case we assume that . This is true in the JAK-STAT and the NF-*κ*B example. Here the complexity of the eigenvalue method and the orthogonal method reduce to  and , respectively. In this case the eigenvalue method is an order of magnitude in the number of parameters faster than the orthogonal method.

### F: Correlation is not equal to linear dependence

In order to show that a correlation of ± 1 between two vectors does not necessarily imply their linear dependence, we present a simple counter-example. Consider the vectors *x *= (1, -1)^*T *^and *y *= (2, 0)^*T*^. According to equation (13) the correlation *corr*(*x*, *y*) = 1 results. Clearly, however, *x *and *y *are linearly independent. A set of parameters that are found to be unidentifiable with the correlation method may therefore contain false positives.
